# Information under lockdown: A content analysis of government communication strategies on Facebook during the COVID-19 outbreak

**DOI:** 10.1016/j.heliyon.2023.e15562

**Published:** 2023-04-18

**Authors:** Gal Yavetz, Noa Aharony

**Affiliations:** Department of Information Science, Bar-Ilan University, Israel

**Keywords:** Social media, Government information, Content analysis, COVID-19, Facebook

## Abstract

The aim of this study is to investigate how central government agencies used social media to communicate and disseminate information to the public during the Coronavirus outbreak. In addition, this study seeks to examine the characteristics of the messages, content, and engagement rates of the information which has been published by the four central government bodies responsible for the management of the emerging crisis in Israel. This article is based on content analysis to examine the work of four main government ministries on Facebook (Ministry of Health, Ministry of Defense, Ministry of Education and Ministry of Finance) throughout the first six months of the crisis from February to July 2020. Findings indicate that The Ministry of Health's posts yielded the highest engagement rates compared to the rest of the ministries. Also, we categorized the posts into four main categories: Policy, Advocacy & COVID-19 additional information, Restrictions, Guidelines & Recommendations. In terms of Sentiment analysis, posts containing restrictions have created the highest emotional reactions (positive and negative). Research findings can be deduced regarding modes of distribution in terms of messages, publication dates, and types of content to improve users' responses in terms of audience engagement and sentiment.

## 1 Introduction

The Coronavirus pandemic has brought new difficulties and challenges for the digital public sphere around the world. The rise of information needs among citizens included the ongoing need for data and guidelines regarding livelihood, education, health, and government affairs [[1], [2], [3], [4]].

Social distancing measures, including national lockdowns, emphasized the need for information and digital services [[5], [6], [7], [8]]. This need was expressed in repeated requests from citizens to official and formal bodies such as government ministries to acquire professional and reliable information [[9], [10], [11], [12]]. Government ministries used social media at the height of the pandemic to disseminate information about rumor refutations, real-time community management, information about readiness, protection, preventive measures, and reduction of exposure to the virus and its morbidity [6,9,13]. In Israel, government bodies have begun advocacy and education measures from early February 2020, even before the first confirmed patients were discovered in the country [10,14].

This study examines how the four leading government ministries at the forefront of the crisis in Israel used social media from the discovery of the first Israeli patient in February until the alleviation of the “first wave” at the end of July. Our focus on these four ministries: the Ministry of Health, the Ministry of Education, the Ministry of Finance and the Ministry of Defense, is based on the fact that they were considered as the central executive authorities in managing and facing the crisis in Israel [15]. Thus, the current study seeks to explore the various content distributed by the four leading government ministries on Facebook. The study includes an analysis and comparative examination of the government agencies’ content strategies, focusing on message types, media, and publication times.

The study's main contribution lies in its utilization of a mixed-method analysis that integrates qualitative and quantitative data. This approach provides a comprehensive understanding of how government agencies used social media during the early stages of the COVID-19 outbreak. The findings are valuable as they emphasize the significance of transparent government information, recommend clear messaging, and suggest optimal posting times and content types to enhance audience engagement and sentiment. The current paper is divided into several sections and sub-chapters; The first section is a literature review mainly illustrating the recent body of knowledge regarding government organizations' use of digital tools during crises and emergencies, focusing on the COVID-19 pandemic. The following section presents the research questions and problem definition for the case study of the use of social media by notable government organizations in Israel during the outbreak. The third section describes the research methods, including quantitative and qualitative content analysis. The final section presents the findings in two parts: a descriptive analysis of the quantitative data and a qualitative analysis examining the recurring themes and characteristics presented in the different types of messages.

## 2 Literature review

### 2.1 Government information on social media

The emergence of social media changed the way that the public communicates with government agencies when seeking, creating, and sharing new types of content [16,17].

For example, a content analysis conducted among three large municipal authorities in China examined 4429 different posts by government bodies on social media while identifying seven main categories of government content: Publicity, guidance, information disclosure regarding government affairs, reminders, announcements, citizens interaction, and others [18]. Similarly, DePaula and his colleagues [19,20] priorly implemented this method of analysis on publications of government bodies throughout the United States and suggested different categories of government content: Information provision, input seeking, online dialogue/off-line interaction, symbolic presentation and marketing and changed accordingly to the government's goals.

Governments' social media goals may vary and can include increasing transparency, strengthening citizen participation, and building collaborations [[21], [22], [23]]. However, the mere adoption and launch of digital representations for government organizations are not enough to achieve these goals, as they might alter according to cultural differences [24,25]. Moreover, social media usage might be influenced by socio-demographic factors such as place of residence, income, education, and perception of technology as a platform for social change [26]. Communities' perceptions regarding public bodies and their work in digital spaces greatly impact governments' choice of communication [27]. Further, researchers have found a connection between the organization's image and self-presentation on social media and public attitudes toward them [28]. In addition, several researchers proposed that government ministries that want to strengthen their images and public engagement and discourse with citizens on social networks should promote and create unique and tailored content for relevant communities [29]. For example, during the COVID-19 outbreak in Israel, it was suggested that targeted interventions by the government could improve compliance with physical distancing measures among the Arab population [30]. In a similar manner, Shomron and David [31] stated that responsible risk communication through digital channels and adaptation to cultural media habits helped promote safer behavior during times of emergency for the ultra-orthodox Israeli communities.

In exceptional cases such as emergencies and natural disasters, government bodies often have to quickly adapt their communication strategies in order to effectively address the situation at hand [32]. The strategy employed by a government body may vary depending on how they perceive their relationship with residents, whether as customers, partners, or citizens [33]. This perception has an impact on the organization's message and content strategies, with the goal of producing dialogue or conveying information and messages instantly [33]. The organization's strategy in social media does not only determine what messages or content will be conveyed but also the way messages will be conveyed and the verbal and communicative style in which they are disseminated, whether they contain official, negative, positive, or personal messages [34,35].

Government communication strategies may differ from each country depending on their main policies regarding different situations; however, there are still several principles that should not be overlooked [36]. For example, an effective communication strategy should include coherent messages delivered via suitable platforms and should involve ongoing community engagement with diverse groups [36]. Another example might be the case of the Zika outbreak in 2016 and the use of the Brazilian government's communication strategy regarding its contingency [37]. The research suggested that emphasizing attributes favorable to government officials negatively affected user engagement.

As another example of the importance of government communication strategies, during the California wildfires of 2017, government leaders and organizations were mainly focused on preparedness and neglected relief and recovery information, as was reflected on social media accounts [32]. However, according to Vera-Burgos & Griffin-Padgett [38], leaders who used restorative rhetoric on social media during national crises promoted better transition into the recovery phase, as was the case of Huston, Texas leadership during Hurricane Harvey in 2017. Recent studies have even suggested that the type of media transmitted on social networks by government organizations is related to the types of engagement with users [[39], [40], [41]]. In another study that focused on examining 14,742 Instagram posts of Andalusia's (Spain) government, a clear relationship was revealed between media type and engagement type. Image-based posts generated more likes, while video-based posts generated more comments [41]. A similar finding also emerged in Lappas et al.’s study [40], which examined the Facebook use of five major local government authorities in Greece and found that video-based posts are positively related to a greater number of comments. Also, rich media posts such as videos and images often yield more users' engagement than text-only content [40]. A similar conclusion also appeared in a study that focused on Twitter, suggesting that the use of videos and images resulted in higher rates of users' engagement, such as retweets, favorites, or replies [39].

For example, Lin [42] posited that posts containing images or videos generated more positive reactions, like love, than posts containing text or a link. Different users' reactions trends vary between events and dates and depend on different types of content [42]. Also, posts publications time was found to affect users’ engagement and sentiment [43]. For example, recent studies have shown that posts published on weekends have higher engagement rates than on weekdays, and posts published in the evening yield higher rates than those published during the workday [[43], [44], [45]]. A possible explanation for the differences between the publication times is that users are more available to use their social media in their leisure time, such as after-work hours and on weekends [44].

Overall, different content strategies can contribute to users' responses, engagement, and sentiments [46,47]. Currently, Facebook enables to classify of the types of audiences sentiments by distinguishing between different types of possible reactions, such as positive sentiments, for example (Love, care, laughter, and wow) or those that carry a negative sentiment: (Angry, sad) [42,48]. Ross et al. [49], who studied users’ reactions on Facebook to national terrorist incidents in Berlin, London, and Stockholm, claimed that in national emergencies, the use of more emotional reactions such as love or sadness increases during and after the events.

### 2.2 COVID-19 and government social media

The Coronavirus pandemic onset in 2020 formed new difficulties and challenges for citizens worldwide, creating new information needs regarding livelihood, education, health, and government affairs [1,3,50]. Accordingly, public sentiment and interest have changed through the progression of the pandemic. For example, it was found that Macao citizens' digital behavior changed significantly during the ongoing crisis period [5]. These changes mainly affected citizens' engagement, which escalated in the severe stage of the crisis, with the first dramatic increase in the number of patients and a decrease in the chronic stage of the crisis. In addition, during the acute period of the pandemic, reactions' rate with positive sentiment was the highest, while most government contents dealt with social distancing measures, restrictions, and national lockdown [5]. This trend was echoed in work by Barkur et al. [51], who proclaimed that citizens’ reactions to lockdown messages were mostly positive, while reactions containing negative sentiments such as sadness or anger appeared minorly at this stage. Górska et al. [52] proposed that a similar communication strategy was found to be common and popular by municipal authorities in Poland at the height of the pandemic. The researchers characterized these types of rhetorical messages as a “*We are together*” communication strategy, including focusing on the community and its solidarity.

Further, Wang et al. [6] studied Twitter use by 67 different health and government organizations in the United States through the first months of the COVID-19 onset. They have established an information distribution typology based on a sample of 13,598 relevant tweets regarding the pandemic and its ramifications. Their typology is based on key prominent themes: Strategies and guidance, closures, operations, external resources/knowledge, and more related topics. Tweets that referred to situational information and external resources/knowledge, which direct users to external media sources, were the most frequent messages published.

The use of social media to transmit information and to communicate with citizens during national emergencies was intensified and expanded to many government institutions during the outbreak of 2020 [6,13,53]. Chen et al. [13] examined the use of social media by government authorities in China through an in-depth study and created four different categories of information, which include: (a) Latest news about the COVID-19 crisis. (b) Appreciation for front-line emergency services. (c) Guidance for stakeholders. (d) Information about the government's handling of the crisis. In their study, it was evident that posts that contained the latest news and information about the government's handling of the crisis were the most engaging types of content [13]. Similar findings were also presented in a study that examined the use of another social media platform, TikTok, by government health organizations in China, revealing that video posts that were related to the latest news generated the highest number of comments [54]. In a similar manner, Zhu et al. [7,8] reported that based on a corpus of 25,024 posts on 17 official Weibo accounts in China, the three high-frequency post topics were: terminology, policy initiatives, and infection prevention and control.

### 2.3 COVID-19 and the Israeli government

Because of the global nature of the current crisis, its effects on social media use and information needs are limitless and affect an unprecedented number of countries, cities, communities and citizens worldwide [3,55]. One of which is the case of Israel, which began with lockdown and other social distancing measures and closures early in February 2020 and entered a cross-country lockdown in March [10]. Lack of information during the outbreak, and especially during lockdowns, led to a reported increase in public stress, anxiety, and even a distorted perception of time [10,56,57]. The growing need for information, support, and communication led many Israeli citizens to approach social media channels to help fulfill it [10,56,58]. According to Gesser-Edelsburg et al. [10], the majority of Israeli citizens turned to official information from government organizations about the virus and its ramifications, reporting that the Ministry of Health website was a primary source for information [10].

The use of digital media to communicate and transfer government information to citizens was significantly high during the Coronavirus outbreak days compared to routine times [3,59]. However, as can be seen in previous studies, various government organizations in Israel have already established the use of new digital platforms in order to disseminate information [[60], [61], [62], [63], [64]]. Regardless of the Coronavirus crisis, in recent years, many government organizations in Israel have adopted the use of social networks for the purpose of disseminating information to citizens and have also implemented many practices designed to increase engagement through the use of visual content (videos/photos), and up-to-date information about policy and operations [65,66].

## 3 Problem statement and research goals

The literature review presents a variety of government communication strategies regarding emergencies on social media [6,38,53]. Still, the communication between the public and government agencies is not fully understood as different contingencies and crises continuously emerge. Additionally, we identify a gap in the literature related to the different messages that government agencies implemented during the outbreak and the different users’ sentiment and response towards it. Hence, the main objective of this study is to achieve a comprehensive perspective of the effectiveness of different content strategies by four main government agencies during this crisis. In order to do so, this study examines how different central government agencies used social media, specifically Facebook, in order to disseminate information to citizens about the Coronavirus outbreak.

The study focuses on four government ministries’ social media use: the Ministries of Health, Education, Finance, and Defense, which defined as the main executive authorities responsible for managing the crisis. The study seeks to analyze the content and characteristics of the messages, as well as the engagement rates of the information published by these government bodies.

This research aims to understand the modes of distribution and types of content that were the most effective in terms of audience engagement and sentiment in order to improve citizens’ response to government information during the pandemic. Thus, the following questions and hypotheses arise:

RQ1 What are the differences between the four offices (Ministry of Health, Ministry of Education, Ministry of Defense and Ministry of Finance) in terms of engagement rates (likes, comments, shares, and reactions)?

RQ2 What are the differences between posts that were published during weekdays compared to posts published during weekends?

Prior studies demonstrated that posts that were published on weekends yielded higher engagement rates [[43], [44], [45]]. Hence, we hypothesize that:

**(H1**): *Posts that were published during weekends will yield more engagement rates (in terms of likes, comments, and shares).*

In addition, it was also found that people using social media during a time of leisure (such as weekends or evenings) will demonstrate more positive interaction online and higher engagement rates [44]. Therefore, we predict that:

(**H2)**: *Posts that were published during weekends will yield more reactions with a positive sentiment than on weekdays. Also, posts that were published during weekdays will yield more reactions with a negative sentiment.*

Further, **(H3):** *Posts that are published during nighttime will yield higher engagement rates (in terms of likes, comments, and shares).*

RQ3 What are the differences between the reactions and engagement of posts that were published during nighttime compared to posts published during the daytime?

Similarly, Fähnrich et al. [43], illustrated significant differences between posting schedules in terms of engagement rates in favor of night and evening, also, Gálvez-Rodríguez et al. [67], have demonstrated a positive connection between nighttime posting and positive sentiment. For all these reasons, we can assume the following:

**(H4):** *Posts that are published during nighttime will yield more reactions with a positive sentiment than daytime posts.*

RQ4 What are the differences in terms of engagement and reaction to posts that contain visual content compared to plain textual posts?

Government agencies are increasingly using visual content on social media platforms, such as images and videos, as they have been found to generate higher rates of engagement among audiences [[39], [40], [41],^66^], and higher rates of positive sentiment [42].

Hence, we hypothesize that:

**(H5):** *Posts that contain richer types of visual media (photos and videos) will yield higher engagement rates (in terms of likes, comments, and shares) compared to plain textual posts.*

(**H6):** *Posts that contain richer types of visual media (photos and videos) will yield more reactions with a positive sentiment compared to plain textual posts.*

The following research questions, RQ5 and RQ6, do not include hypotheses as they are exploring novel and specific aspects of the Israeli government and political system in relation to the COVID-19 outbreak.

RQ5 What are the main types of information and content published on Facebook by the four offices during the COVID-19 outbreak?

RQ6 What are the differences between the four identified types of information in terms of engagement and reactions?

## 4 Methodology

In order to answer these research questions and hypotheses, we have chosen the content analysis approach as a leading methodology. Content analysis provides us with many insights about texts, messages, and various pieces of information which can be examined within the context in which they were created and produced. Content analysis strengthens our understanding of the phenomena being studied and offers us new interpretations of the phenomenon under examination [68]. Content analysis can be based on counting quantitative variables but also incorporate qualitative analysis to enrich insights and identify patterns. Content analysis can involve both quantitative and qualitative approaches [69]. While quantitative methods focus on counting and measuring variables such as words, news articles, and messages, qualitative techniques can add richness and depth to the analysis by identifying patterns and themes [70]. When conducted systematically, qualitative content analysis can produce insightful interpretations of the text or subject matter, highlighting prominent categories and emerging themes [[70], [71], [72]]. As prior research has shown, using a qualitative approach can be beneficial for studying different aspects and case studies on social media [73]. Such an approach can be useful, for instance, in investigating government content on social media platforms [5], political messages of state leaders [62], and for the use of crisis communication in times of emergencies, when a profound understanding of agendas and messages is needed [74].

The focus of this study will be to review posts posted on Facebook by the four government ministries at the center of this study. Our choice of Facebook as a major and leading platform for research stems from a number of assumptions. First, despite the diverse use of social networks, Facebook is still the most common among Israeli citizens, by 81% of adults, compared to 52% on Instagram and only 15% on Twitter [75]. Second, despite the varied use of social media between government ministries, Facebook was the only platform shared by these four ministries during the period under review and in general.

### 4.1 Procedure

The information and data from Facebook were extracted using CrowdTangle software, which is owned by Facebook. CrowdTangle allows exporting data from public pages, open groups, and verified profiles. The software does not allow the export of data and information about private users or closed groups [76]. In addition, the software allowed us to export all user responses and engagement rates to each public post posted by the pages as likes, comments, shares and the various possible reactions, like positive sentiment replies (love, care, laughter, wow) as well as reactions with negative sentiment (angry, sad) [48]. Thus, we used the search options of the software to export all the posts and engagement rates of the four Facebook pages of the following government ministries: Ministry of Health, Ministry of Defense, Ministry of Finance and Ministry of Education.

### 4.2 Sampling frame

**Fig. 1:**
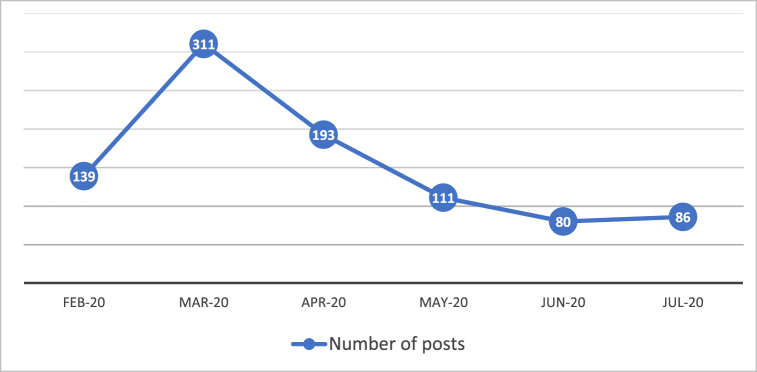
Total number of posts from the four offices during the first six months of the outbreak.

In order to present as relevant and representative a picture as possible, we have chosen to focus on a period of six months from 1.2.2020 to 31.7.2020, as the first six months of the epidemic in Israel. Although the first verified infection case in Israel occurred on February 21, reports began on Israeli passengers on the Diamond Princess cruise ship. A number of verified patients were identified as early as February 5. Thus, the Ministry of Health's information efforts to make information accessible began to increase regarding the spread of the virus and ways of dealing with it [77]. Compared to February, which was devoted by government efforts to build initial awareness among citizens, the following March defines the period of proactive activity, which included isolation measures for returnees from abroad starting on the 9th of that month. Also, a general curfew and a national lockdown were announced a few days later [14]. In parallel with these activities, government ministries turned to digital media in order to make important and relevant information accessible on this subject. Fig. 1 depicts the number of Facebook posts posted by the four government ministries during the six-month period of the outbreak.

As can be seen in Fig. 1, in February, which was defined as the period of defense and preparation for the crisis to come, the total number of posts reached 139 that month. Subsequently, in March, the number of posts published by the ministries reached a record high of 311, which might reflect the increased advocacy efforts that have taken place following and in parallel with active measures such as closures and other mandatory measures. Months April and May presented a clear decline of posts which marked the exit from the general lockdown and the end of the “first wave.” June and July contained almost the same number of posts (80 and 86, respectively), reflecting the sense of ‘return to routine’ presented by the government in those days. A total of 920 posts were published by the four ministries during the half-year examined. Out of the 920 posts, we found that 145 (15.7%) were not directly related to COVID-19. Thus, we focused on 775 posts (N = 775) in total.

### 4.3 Data analysis

The research method and data analysis used in this article are of the type of mixed content analysis: quantitative and qualitative. In the first phase of this study, the four pages were examined comparatively according to the amount and frequency of distribution of the posts between them and according to users' engagement rates. The engagement rates were examined according to the following metrics: likes, comments, shares, and reactions with a positive or negative sentiment. In addition, we tested the correlations between the levels of engagement and other variables, such as the time of publication (time of day and day of the week), media type, and content category. The distinction by the time of day was made dichotomously by posts published between 06:00 a.m. and 17:59 p.m. and posts published between 18:00 p.m. and 05:59 a.m. The day-of-the-week comparison was made between weekends and weekdays following the Israeli workdays from Sunday to Thursday (weekdays) and Fridays and Saturdays (weekends). The classification of content categories was done manually by two of the authors, with one researcher creating the first categories according to the leading content of each post (n = 775) and a second coder performing a double coding of 33% of the sample (n = 260). Inter-coder reliability (Krippendorff's alpha index) for content categories typology was .86 and is considered reliable [78].

Differences between several groups on study variables were examined via the Kruskal-Wallis H test. Post hoc tests with adjusted p-values (i.e., Dunn test with Bonferroni correction) were conducted to evaluate pairwise differences. For comparisons between two groups on research variables, a Mann-Whitney test was conducted. All data analysis was conducted using IBM SPSS statistics version 26. An alpha level of 0.05 was used for all statistical tests.

### 4.4 Coding schemes

The second dimension of coding dealt in depth with the coding and characterization of all the posts in the sampling according to the main topic, the main theme in which they dealt. Table 1 describes all categories and their description in the way they were coded by the first researcher inductively and by the additional researcher deductively. The original categories were driven by the suggested framework of Chen et al. [13], with localized adaptation.

**Table 1:** Coding scheme for content category.

Content Category	Description
Advocacy and COVID-19 additional information	Providing education and information about the virus, the disease, and its symptoms and debunking misinformation about COVID-19 in general.
Restrictions	Public announcements regarding the closing of services, business, lockdowns, curfews, and distance limitations.
Guidelines and Recommendations	Recommendations regarding maintaining an active and healthy lifestyle during the crisis and practical ways to avoid and reduce exposure to the Coronavirus.
Policy	Information about the office activities and policies (e.g., laws and regulations regarding financial assistance, epidemiological investigations, public events (virtual or physical) organized by the office and its staff.

## 5 Results

Table 2 presents medians, means, and standard deviation of engagement (Likes, comments, and shares) and reaction rates (positive and negative sentiment) by office page.

**Table 2:** Engagement and reaction rates by office page (N = 775).

Variable		Office page				
		Education	Health	Defense	Finance	
		**(*n* = 332)**	**(*n* = 252)**	**(*n* = 105)**	**(*n* = 86)**	***H*(3)**
Likes	*Md*	57.00_b_	299.50_a_	273.00_a_	36.00_c_	357.07***
	*M*	107.51	643.17	412.96	51.41	
	*SD*	199.95	1244.55	723.68	60.11	
Comments	*Md*	9.00_c_	154.50_a_	28.00_b_	25.50_b_	298.40***
	*M*	38.48	306.44	48.29	63.58	
	*SD*	79.51	422.47	60.51	162.09	
Shares	*Md*	11.00_c_	78.00_a_	30.00_bs_	7.50_c_	217.81***
	*M*	28.48	266.03	58.93	18.71	
	*SD*	80.32	836.06	132.30	32.65	
Positive Sentiment	*Md*	2.00_c_	11.00_b_	20.00_a_	1.50_c_	206.48***
	*M*	9.44	38.29	36.85	4.67	
	*SD*	40.40	83.87	59.67	10.67	
Negative Sentiment	*Md*	0.00_c_	7.00_a_	0.00_b_	0.00_b_	263.08***
	*M*	3.86	21.71	23.06	7.02	
	*SD*	13.97	42.39	121.47	35.00	

*Note*. Medians sharing a common subscript are not statistically different at α = 0.05 according to Dunn test with a Bonferroni correction.

***p < .001.

As the data shows, the Ministry of Education published the highest number of posts regarding the Coronavirus during the examined period, while the Ministry of Health ranked second. Comparing the four offices on engagement rates yielded significant results for all measures. Pairwise comparisons revealed that the Ministry of Health (MOH) yielded more likes than the ministries of Education (MOE) and Finance (MOF), but not different from the ministry of Defense (MOD). Similarly, the MOD yielded more likes than the MOE and MOF. The MOE yielded more likes than the MOF. Regarding comments, the MOH yielded higher rates than all other offices. The MOD and MOF yielded more comments than the MOE, but were not different from each other. With regard to shares, the MOH yielded higher rates than all other offices. The MOD yielded more shares than MOF and MOE. Lastly, the difference between the MOF and the MOE was non-significant.

Analyzing office differences in reactions with positive sentiment showed significant results, indicating that the MOF yielded less positive sentiment than MOH and MOD, but was not different from MOE. The MOE yielded the same pattern of results. Finally, the rates of positive sentiment were higher for the MOH as compared to the MOD (Table 1).

In addition, MOH yielded more reactions with negative sentiments than all other offices. All differences between the MOD, MOF, and MOE were non-significant (Table 1).

Examining the differences between posts that were published during weekdays (*weekdays*) and weekends (*weekend*s) on engagement rates (H1), revealed significant results with *weekends* generating more likes, comments, and shares than *weekdays* (Table 3).

**Table 3:** Engagement and reaction rates by day of the week (N = 775).

Variable		Day of week		
		Weekdays	Weekend	
		**(*n* = 672)**	**(*n* = 103)**	***U***
Likes	*Md*	100.00	150.00	29665*
	*M*	317.83	310.40	
	*SD*	847.37	478.90	
Comments	*Md*	26.00	85.00	25342***
	*M*	120.98	186.78	
	*SD*	277.13	301.80	
Shares	*Md*	19.00	38.00	28651**
	*M*	107.83	114.88	
	*SD*	516.54	309.87	
Positive Sentiment	*Md*	5.00	7.00	31644
	*M*	22.35	19.78	
	*SD*	62.57	47.07	
Negative Sentiment	*Md*	3.00	1.00	26081***
	*M*	16.05	12.09	
	*SD*	40.33	55.24	

*p < .05 **p < .01 ***p < .001.

Differences between weekdays and weekends on reactions with positive or negative sentiment (H2) showed significant results only for negative sentiment, indicating higher rates of reactions with negative sentiment during weekends compared to weekdays (Table 2).

Regarding the hypothesis about the differences between posts that were published during daylight (daytime) and nighttime (nighttime) on engagement rates (H3), analyses yielded significant results for all measures. That is, nighttime yielded more likes, comments, and shares compared to daytime (Table 4).

**Table 4:** Engagement and reaction rates by time of publishing (N = 775).

Variable		Time of publish		
		Daytime	Nighttime	
		**(*n* = 556)**	**(*n* = 219)**	***U***
Likes	*Md*	91.00	138.00	49592***
	*M*	240.24	511.34	
	*SD*	409.71	1355.39	
Comments	*Md*	26.00	51.00	48108***
	*M*	97.18	212.34	
	*SD*	230.12	369.34	
Shares	*Md*	19.00	32.00	51709**
	*M*	75.48	193.26	
	*SD*	194.46	871.73	
Positive Sentiment	*Md*	4.00	7.00	50014***
	*M*	15.73	37.95	
	*SD*	34.22	98.76	
Negative Sentiment	*Md*	1.00	2.00	54779*
	*M*	8.65	22.68	
	*SD*	26.56	90.68	

*p < .05 **p < .01 ***p < .001.

Analyzing differences between *daytime* and *nighttime* on reactions with positive or negative sentiment (H4) showed significant results for both sentiments, such that *nighttime* yielded more reactions with a positive or negative sentiment than *daytime* (Table 3).

Analysis of differences between posts containing photos (*photos*), *videos* (videos), and plain text (*text*) on engagement rates (H5) showed significant results for all measures (Table 5).

**Table 5:** Engagement and reaction rates by category of post (N = 775).

Variable		Type			
		Photo	Video	Text	
		**(*n* = 442)**	**(*n* = 244)**	**(*n* = 89)**	***H*(2)**
Likes	*Md*	97.00_b_	112.50_ab_	145.00_a_	13.76**
	*M*	230.51	464.51	340.80	
	*SD*	380.74	1296.66	522.70	
Comments	*Md*	26.00_b_	32.00_b_	88.00_a_	29.10***
	*M*	106.90	150.25	186.79	
	*SD*	253.37	330.22	256.29	
Shares	*Md*	17.00_b_	25.00_a_	32.00_a_	16.27***
	*M*	83.30	154.39	110.12	
	*SD*	218.37	813.39	266.11	
Positive Sentiment	*Md*	4.00_b_	10.00_a_	5.00_b_	41.74***
	*M*	13.73	38.51	17.84	
	*SD*	48.50	81.21	36.28	
Negative Sentiment	*Md*	0.00_b_	1.00_b_	5.00_a_	39.77***
	*M*	7.83	18.25	20.93	
	*SD*	22.77	84.95	48.66	

*Note*. Medians sharing a common subscript are not statistically different at α = 0.05 according to Dunn test with a Bonferroni correction.

**p < .01 ***p < .001.

Pairwise comparisons revealed that *photos* yielded fewer likes than *text*, with both types of posts not differing from *videos*. Regarding comments, *photos* and *videos* yielded lower rates than *text*, but were not different from each other. With regard to shares, *photos* yielded lower rates than *text* and *videos*. The difference between *text* and *videos* was not significant (Table 4).

Differences in reactions with a positive sentiment or negative sentiment by type of post (H6) showed significant results for both measures, indicating that *videos* yielded more positive sentiment than *photos* and *text*. The difference between *photos* and *text* was not significant. Regarding reactions with a negative sentiment, *photos* and *videos* yielded lower rates than *text*, but were not different from each other (Table 4).

**Fig. 2:**
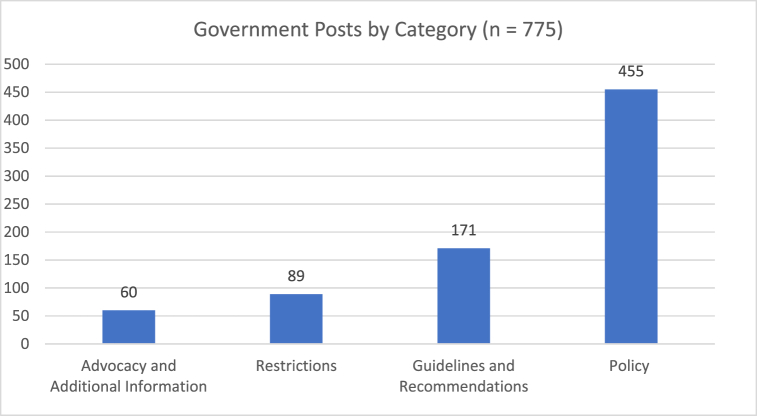
Distribution of content categories for all pages.

The fifth research question (RQ5) sought to examine the main themes for the government posts. Fig. 2 depicts the distribution of posts by content categories for the four Facebook pages.

### 5.1 Policy

Posts belonging to this category appeared most frequently (n = 455) out of all posts reviewed (58.7%). An in-depth analysis of the posts shows that some of the posts in this category dealt with epidemiological investigations and mainly came upon the Ministry of Health page and sometimes on the Ministry of Education page. For example, the following post which was published by the Ministry of Education on 23-2-20:

“After mapping the school’s routes, 30 students in Be’er Sheva were found to be exposed to the group and therefore went into isolation. At the moment, none of them have symptoms […] We work to prevent any possible exposure to the virus, and we are determined to continue to maintain a complete and normal study routine in every possible scenario."

A different type of post belonging to that category contained information about the activities of the various ministries and the announcement of a new policy regarding the crisis, such as compensations, grants, or regulations. An example can be seen on the page of the Ministry of Finance, as it was published on 27-03-20:

“A special grant of up to NIS 6,000 for those aged 67 and over whose work was terminated due to the corona crisis, in addition to their entitlement to the pension; the full regulations will be published on the National Insurance website."

### 5.2 Guidelines and recommendations

The second most common category (n = 171) dealt with the guidelines and recommendations regarding the Coronavirus. Thus, posts that belonged to this category contained information regarding recommendations for dealing with the virus, reducing exposure, or maintaining an active and healthy lifestyle during the crisis. An example of these guidelines can be seen on the Ministry of Health page at the beginning of the crisis in February in one of the first posts that dealt with COVID in general:

“Have you returned from China in the last 14 days? Pay attention to the new Ministry of Health guidelines for returnees from China! Even if there are no signs of illness, the guidelines are valid for the first 14 days of return, which is the time of incubating the Coronavirus" (2-2-20)

Another type of post from that category addressed the guidelines and recommendations regarding the education system in Israel and was published by the Ministry of Education in the early days of the first outbreak:

*“Dear parents, these days, you are facing a new reality related to the spread of the coronavirus in Israel. This is an unfamiliar reality that involves uncertainty. One of the guidelines for preventing the spread of the virus is home isolation. We have attached a number of recommendations and activities for your child's quarantine”* (09-3-20)

### 5.3 Restrictions

The third category of posts appeared in 89 posts which constituted about 11.4% of the sample. These posts contained information regarding various restrictions: closure of educational institutions and public institutions and mandatory social restrictions in general. A prominent example of this type of post, which embodies a number of limitations, can be seen on the page of the Ministry of Health, which was published on 22-3-20:

*“Leaving the house is possible only in special cases! The Israeli government has approved emergency regulations. The regulations require every person to stay at home, except in special cases […] Also, a two-meters distance between people is required in public space, and travel of more than two people in a vehicle is prohibited […] We all are required to comply with restrictions*"

Despite the majority of restrictions addressing the national crisis, some ministries mentioned specific restrictions relating to their own activities, as in the case of the Ministry of Defense:

*“Due to the outbreak of the coronavirus, and out of concern for the health and safety of the disabled in the IDF, there will be no reception in the rehabilitation districts throughout the country, in the medical committees, and in the headquarters units. This will start tomorrow - until further notice”* (15-3-20)

### 5.4 Advocacy and COVID-19 additional information

Posts that belonged to this category contained extensive explanations, education, and data. Posts of this type appeared at the lowest frequency (n = 60) and accounted for only 7.7% of the total sample examined. Most of the posts in this category contained references to online conversations with physicians, as the following post by the Ministry of Health:

*“When it comes to the Coronavirus, it is important to get the information from authorized sources. Click and watch doctors answer questions you have asked on the subject. For all real-time updates, go to the Ministry of Health Telegram channel.”* (12-3-20)

This type of content mostly addressed Q&A (Questions and answers) sessions, as one of them, referred to by the Ministry of Defense:

“From now and until 12 pm, we are here to answer all your concerns, questions and inquiries about the coronavirus. Write down your question in the comments section below and we will try our best to address all of your concerns" (13-3-20)

Another type of post in this category provided specific information regarding the commissioning of rumors and misinformation about fake news published on the Internet. An example of such a post can be seen on the Ministry of Health page, as it appeared on 12-2-20:

“Lots of rumors circulating on the net about the Coronavirus helped us spread accurate information.”

Table 6 presents medians, means, and standard deviation of engagement and reaction rates by the four posts category.

**Table 6:** Engagement and reaction rates by category of post (N = 775).

Variable		Category				
		Policy	Guidelines	Restrictions	Advocacy	
		**(*n* = 455)**	**(*n* = 171)**	**(*n* = 89)**	**(*n* = 60)**	***H*(3)**
Likes	*Md*	90.00_c_	124.00_bc_	282.00_a_	173.50_ab_	36.36***
	*M*	183.09	332.49	877.71	454.65	
	*SD*	254.05	524.70	2039.32	661.23	
Comments	*Md*	24.00_c_	35.00_bc_	141.00_a_	74.50_ab_	51.38***
	*M*	71.03	138.88	343.56	231.53	
	*SD*	145.60	250.30	514.39	439.14	
Shares	*Md*	17.00_c_	24.00_b_	80.00_a_	37.50_ab_	55.98***
	*M*	46.03	124.85	401.18	104.93	
	*SD*	114.50	263.42	1343.70	215.79	
Positive Sentiment	*Md*	4.00_b_	5.00_b_	15.00_a_	7.00_ab_	28.37***
	*M*	13.38	23.46	61.31	24.95	
	*SD*	27.25	73.16	120.85	50.35	
Negative Sentiment	*Md*	0.00_b_	1.00_b_	8.00_a_	2.00_b_	47.05***
	*M*	7.97	7.67	50.91	5.15	
	*SD*	23.92	30.80	136.52	9.86	

*Note*. Medians sharing a common subscript are not statistically different at α = 0.05 according to Dunn test with a Bonferroni correction. Guidelines = Guidelines and recommendations; Advocacy = Advocacy and COVID-19 additional information.

***p < .001.

Comparing the four categories of posts on engagement rates yielded significant results. Pairwise comparisons revealed that posts containing information about restrictions (*restrictions*) yielded more likes compared to posts containing information about policy (*policy*) or guidelines and recommendations (*guidelines*). *Advocacy* yielded more likes than *policy*. Exact patterns of results were obtained for comments. That is, *restrictions* yielded more comments compared to *policy* or *guidelines* but not compared to *advocacy*. *Advocacy* yielded more comments than *policy*. With regard to shares, *restrictions* yielded higher rates than *guidelines*, and *policy* yielded fewer comments than all other categories. No other significant differences were found among the types (Table 5).

Analyzing differences in reactions with a positive sentiment or negative sentiment by category of the post showed significant results for both measures, indicating that *restrictions* yielded more positive sentiment compared to *policy* or *guidelines* but not compared to *advocacy.* Regarding reactions with a negative sentiment, *restrictions* yielded higher rates than all other categories. The remainder of the comparisons showed non-significant results (Table 5).

## 6 Discussion and conclusions

The study focused on four government ministries that were perceived as central during the COVID-19 crisis and on their different communication strategies. The first research question (RQ1) sought to examine and focus on the differences between the four ministries in terms of users' engagement rates. In order to answer it, we examined the differences according to the types of engagement (likes, shares, comments) and the post's source. Our findings reinforced prior work by Gesser-Edelsburg et al. [10], who found that the Ministry of Health was perceived as the most significant factor in health information acquiring for Israeli citizens during the Coronavirus crisis. As our findings have shown, the Ministry of Health posts yielded the highest rates of both negative and positive reactions. A possible explanation for this might lie in the emotional and actual nature of the office's content regarding the spread of the disease, infection rates, and hospitalizations through the outbreak in Israel [10]. This finding also reinforces prior studies that showed a connection between the governmental body's centrality in managing the COVID-19 crisis and the types of reactions of citizens to it [6]. This is eminently relevant in our case since the Ministry of Health was considered the primary governmental and regulatory body that dealt with the crisis in Israel [79].

The second research question (RQ2) sought to examine the differences between posts that were published during weekdays compared to posts published during weekends. In order to answer this question, the following hypotheses were examined. H1 assumed that posts that are published during the weekend would yield higher engagement rates than those published during the workweek. This hypothesis was fully supported and is consistent with previous work [[43], [44], [45]], suggesting that it can be explained by individuals’ availability to use social media. Similarly, our second hypothesis assumed that posts that are published on weekends would receive reactions with more positive sentiment than posts published during the week. This hypothesis was also fully supported. A possible explanation for this can be found in the fact that in these times of leisure, people feel free from their daily jobs or travel and, therefore, already have a more positive approach [44,45]. The third research question (RQ3) focused on the aspect of the date of publication in terms of differences in engagement rates and types by nighttime or daytime. In regard to this issue, our two hypotheses have reinforced prior findings as they indicated that citizens would be more available to use social media than during the workday, and this also can affect their mood and attitude [[43], [44], [45]]. Thus, posts that were published during times of leisure yielded higher rates of engagement and positive sentiment.

The fourth research question (RQ4) sought to examine the differences in terms of engagement and reaction to posts that contain visual content compared to plain textual posts.

In accordance with this question, two research hypotheses concerning the relevant variables were examined. H5 assumed that posts containing richer types of visual media (photos and videos) would yield higher engagement rates (in terms of likes, comments, and shares) compared to plain textual posts. To our surprise, and contrary to our hypothesis and previous findings in the literature [[39], [40], [41],^66^], text-based content yielded higher engagement rates than visual content. A possible explanation for these findings is that the percentage of textual posts was higher in the Ministry of Health, which was found as the ministry with the highest engagement rates significantly compared to other ministries. H6 assumed that posts that contain richer types of visual media (photos and videos) would yield more reactions with a positive sentiment, compared to plain textual posts. This hypothesis was fully supported and reinforces Lin's [42] previous findings.

The fifth research question (RQ5) sought to examine in a comparative and in-depth manner the main types of information and content published on Facebook by the four offices during the COVID-19 outbreak. Using a thematic analysis, we found that the posts relevant to the Coronavirus crisis dealt with four main categories: Policy, Advocacy and COVID-19 additional information, Restrictions, and Guidelines and Recommendations. The majority of posts were coded as *Policy* (58.7%), while *Guidelines and Recommendations* appeared next in frequency (22.2%). *Restrictions* and *Advocacy* were the least common categories (11.4% & 7.7%, respectively). Our findings also indicated that posts containing information about restrictions yielded more engagement rates than the other categories by all measures (likes, comments, shares) (RQ6). This finding is in line with prior work by Pang et al. [5] that found a similar pattern in the case of social media use by Macao County during the COVID-19 outbreak in early 2020. In addition, *Restrictions* based posts were found to yield the highest rates both for positive and negative reactions. This finding is somewhat in line with prior work by Barkur et al. [51], indicating that information about the lockdown and other mandatory measures yielded mostly positive sentiment by citizens during the outbreak. Our findings illustrate that information about restrictions generates the highest emotional reactions, both positive and negative sentiments.

## 7 Implications, limitations, and recommendations

Our findings suggest that government agencies should better acknowledge the value of transparent and updated information about mandatory measures during crises in order to recruit more public support online. They should also consider the effectiveness of plain, coherent messages to explain those measures further. In addition, practitioners should adhere to posting on leisure time schedule rather than on regular working hours, as the attention span might be improved. This work is not without its limitations; First, our study is based only on Hebrew content, and it is possible to assume that different strategies or textual messages would be interpreted differently due to cultural differences. Furthermore, as this study focuses only on Hebrew-based content, as it is the official language in Israel, one should consider that there are other communities and minorities whose prime language is Arabic, English, or Russian. This content might exclude some from fully participating in the online discourse. Second, our work is based on the first half of 2020, which is described as the first onset period of the Coronavirus. Due to time limitations, we could not investigate the second or third waves of the pandemic. In addition, we would like to recommend a number of future research directions. We recommend conducting further research to get a complete picture, specifically looking at how government organizations used social media in the latter half of 2020 when vaccines became available and changed the nature of the pandemic for some countries. Further, we believe that valuable insights can be achieved by implying the same methods on different case studies of other countries and cultures with their efforts to fight the pandemic.

## Author contribution statement

Gal Yavetz; Noa Aharony: conceived and designed the experiments; performed the experiments; analyzed and interpreted the data and wrote the paper.

## Data availability statement

Data will be made available on request.

## Declaration of competing interest

The authors declare the following financial interests/personal relationships which may be considered as potential competing interests:

Noa Aharony reports financial support was provided by Dangoor Personalized Medicine Fund at 10.13039/501100002744Bar-Ilan University.
